# Prevalence of Orthorexia Nervosa and Its Diagnostic Tools—A Literature Review

**DOI:** 10.3390/ijerph18105488

**Published:** 2021-05-20

**Authors:** Antoni Niedzielski, Natalia Kaźmierczak-Wojtaś

**Affiliations:** 1 Independent General Psychology Unit, Medical University in Lublin, 20-093 Lublin, Poland; n.kazmierczak@o2.pl; 2 Centre of Postgraduate Medical Education, 01-813 Warsaw, Poland

**Keywords:** orthorexia nervosa, tools, prevalence, eating disorders, ORTHO-15

## Abstract

The aim of this article is to present the up-to-date diagnostic tools of orthorexia and markers of its prevalence on the basis of the available literature. The authors searched PubMedCentral (PMC) and Google Scholar with the search entry of “orthorexia”, “orthorexia nervosa”, and “orthorexicbehaviours”. We describe the tools of evaluation of orthorexicbehaviour (i.e., orthorexia self-test—BOT, the ORTO-15 questionnaire, Eating Habits Questionnaire—EHQ, Düsseldorf Orthorexia Scale—DOS, Teruel Orthorexia Scale—TOS, Barcelona Orthorexia Scale—BOS, and Orthorexia Nervosa Inventory—ONI), and offer a review of the studies on orthorexia nervosa. We conclude that there are no reliable data regarding the prevalence of orthorexia nervosa. The available studies point to significant differences in the prevalence depending on the value of cut-off points and tools used. The prevalence varies across countries and across populations, ranging from 6.9% in the Italian population to 88.7% in the group of Brazilian students of dieting. Thus, it indicates that some groups seem to be susceptible to the risk of ON more than others. It is a challenge to determine the prevalence of orthorexia, and any obtained results should be treated with caution. Consequently, we claim that the use of the ORTO-15 questionnaire to diagnose orthorexia is questionable due to a high percentage of falsely positive results.

## 1. Introduction

Recently, a lot of scientific disciplines have witnessed an ever-increasing interest in health and healthy eating habits. Our eating habits have an effect not only on our growth and physical development but also on our fitness and well-being. A healthy diet is a prerequisite of health; it promotes healthy immune system and fosters fast recovery. However, an excessive concentration on food quality may paradoxically be unhealthy.

Orthorexia nervosa (ON) has been subject to more and more studies over the recent years. The term itself was coined by Steven Bratman in 1997, who signalled a potential existence of a new eating disorder. It is defined as a fixation on healthy eating [1] and is characterised by an excessive concentration on food quality, food preparation, and rigorous standards of nutrition norms. 

Those with the symptoms of orthorexia nervosa eliminate products containing preservatives, colour additives, food flavouring, pesticides, excessive fat, sugar, salt, or genetically modified food from their diets [2,3,4]. They rely on foods coming from ecological farming [5,6]. A list of acceptable foods may be subject to individual variation, yet what is characteristic of ON is a gradual intensification of imposed diet ary restrictions. A cause of obsessive thoughts can be the process of food preparation itself (e.g., use of natural materials, preference of earthenware and wooden products over aluminum) or a menu preparation and food purchase [1,6,7,8]. Meals are prepared with the utmost care and attention, and any deviation from the imposed norms leads to a feeling of fear, guilt, shame, and further dietary restrictions [1,6,9].

According to Varga et al. [10], ON can be perceived as a continuum, with one extreme being a healthy diet, and the other one being a pathological interest in healthy eating habits. Bratman [11] observed that two stages could be distinguished in the course of ON development, i.e., healthy orthorexia, with an interest in healthy eating with no pathological features, and orthorexia nervosa, with an obsessive focus on healthy eating. It should be clarified then that a focus on healthy eating is not a disorder per se; however, an excessive fixation on the quality of foods and their preparation, together with negative behavioural consequences, may lead to ON. 

Orthorexia nervosa is not listed in the offical ICD-11 and DSM-V classifications of mental disorders. There is still no officially accepted definition of ON, or standardised criteria of its diagnosis. Even though many diagnostic criteria have been offered [10,12,13,14], all of them have been criticised. In 2016, Dunn and Bratman [15] developed new diagnostic criteria on the basis of their analysis of the published studies, data obtained from experts on eating disorders (from USA, Norway, Poland, Sweden, Australia, Italy, and Germany), and questionnaires. The criteria were divided into A and B type. The former described behaviour characteristic of ON, i.e., obsessive eating habits, feeling of anxiety when not following the dietary restrictions, consequently leading to their intensification. A loss of body mass index was observed in those with ON; however, it was not a necessary and sufficient condition of ON. Criteria B point towards a wide spectrum of ON-related consequences (malnutrition, social isolation, distorted image of one’s body, low self-esteem). It should be stressed here that these criteria still need to be validated and can be subject to further modification [15].

The status of ON as a mental disorder is subject to a discussion. There is no consensus among researchers whether ON should be regarded as a mental disorder, a variety of well-known disorders, or just an unhealthy eating habit [16].

Some researchers highlight the fact that ON shares some of its features with anorexia nervosa (AN). Both ON and AN can be characterised by striving for perfection, high levels of anxiety, and a need to control [2,3,9,17,18]. In both of them, an excessive focus on healthy eating habits can be observed [2,6,19]. However, those suffering from ON focus on the quality of food, while those with AN focus mostly on the quantity of food [1,10,20]. Some researchers claim that fixation on the quality and type of food can be observed in those suffering from AN, since they follow certain strict rules of dieting [16,21]. Therefore, fixation on the quality and type of food may not be the necessary and sufficient condition of ON. A rigid selection and gradual reduction of “acceptable” products can be observed in both of the disorders, yet those with ON limit their diets in order to stay optimally healthy rather than for fear of obesity, typical of AN [1,10,15,22]. Deviations from the eating habits are identified by both groups as a lack of self-control [1]. In the case of ON and AN, symptoms are perceived as egosyntonic, which may diminish the motivation for treatment [2]. Some studies link a significant and purposeful loss of body weight, together with a distorted image of one’s body, only with AN [1,2,23,24]. However, the recent studies seem to demonstrate that there is a correlation between ON and striving for a lowered body weight and distorted self-image and self-esteem [12,22], which would further point to a correlation between ON and AN. Some other studies point out that ON and AN should be treated as a continuum of the same psycho-pathological dimension of various degrees [25,26]. Mac Evilly suggested that ON should be a risk factor and an initial stage of developing an eating disorder (ED) rather than a separate disorder [27]. Eating habits observed in the course of development of ON can become more and more restrictive and compulsive, and consequently lead to an eating disorder. Other studies indicate that ON may be a co-existing disorder or even a strategy to cope with an ED [22,28,29]. A focus on healthy foods and a reduction of the fixation on the intake of calories may paradoxically lead to an increase in the variety of food and lowered risk of losing weight. Even though patients remain selective in their food choices, they start taking in more calories, and it may be a first step towards a recovery after an ED [28].

In the light of the DSM-V classification, ON can becategorised as Avoidant Restrictive Food IntakeDisorder (ARFID) [13,22]. ARFID can be characterised by a lack of interest in food, eating, avoidance of certain types of food (shapes, colours), and being afraid of the consequences of eating [30]. However, anxiety connected with eating may be the result of a traumatic experience (e.g., choking) or an aversive experience (e.g., regular vomiting) [30,31], rather than a mere result of an excessive fixation on health issues. It should be noted that the abovementioned risk factors of AFRID are not exhaustive; therefore, we cannot rule out that ON-like food quality factors or fear of consequences (poor health) may be appropriate and indeed formally endorsed in future versions of ARFID.

Apart from the similarities to ED, ON exhibits some overlap with OCD [20,32,33]. The shared symptoms are obsessive thoughts (e.g., thinking about healthy food, food planning), repeated activities (e.g., preparation of food, weighing of products, checking the etiquettes) [34], and disorders of social functioning and low quality of life [8,33]. However, in contrast to ON, symptoms of OCD are of anegodystonic character [1,9,33].

The treatment of ON does not involve any specific therapeutic approach, since there is no officially accepted definition of ON. The available literature shows that the treatment may be based on a multidisciplinary approach and a team of physicians, psychotherapists, and dieticians [7,35], which allows the combination of the contribution of pharmacology, psychotherapy, and psycho-education [9]. A balanced diet, the aim of which will be to compensate for malnutrition, is recommended as a basis for the treatment. In the case of a significant body weight loss, hospitalisation may be necessary [13]. Cognitive–behavioural therapy is also recommended together with pharmacotherapy and selective serotonin reuptake inhibitors (SSRI) such as fluoxetine, sertraline, and paroxetine [9]. Anti-psychotic drugs, such as olanzapine, can be used in order to alleviate the obsessive character of thinking about food [13]. It should be noted that those with ON may reject drugs as “unnatural” substances [9]. Psychotherapy should not focus only on what patients eat but also on how they do shopping, how they prepare meals, and what they think about their food [35]. Additionally, methods of alterating their eating behaviour may involve enriching their diets and facilitatingsocialising while eating [7]. Relaxation techniques may also be effective in diminishing the anxiety related to eating [36,37,38]. 

Orthorexia is a new phenomenon, and its diagnostic criteria, methods of classification, and basic mechanisms are still being discussed and questioned. It is still not very clear how to diagnose this pathological behaviour and measure the scale of ON, especially becausemany of its symptoms may not exceed the norm or may even be desired. Therefore, the aim of this article is to offer a critical review of the up-to-date diagnostic tools of ON and markers of its prevalence.

## 2. Materials and Methods

The authors reviewed the literature available at PubMedCentral (PMC) and Google Scholar. The searching criteria were as follows: “orthorexia”, “orthorexia nervosa”, and “orthorexicbehaviours”. The review included empirical studies thatrelied on the tools designedfor measuring ON (i.e., BOT, ORTO-15, EHQ, DOS, TOS, BOS, and ONI), and studies which specified the prevalence of ON in a given group of participants. An additional criterion was for the study to be published in a peer-reviewed journal or to be an unpublished PhD dissertation. We excluded studies thatwere not peer-reviewed, commentaries, literature reviews, and duplicated studies (i.e., the same studies published in different languages). Studies published in languages other than English (*N* = 5) were translated. We analyzed studies published beginning from January 2004 (the first publication on ON in a peer-reviewed journal) untilApril 2020. The first step of analysis was the title and abstract, and only then did wefocus on a full text.

## 3. Results

### 3.1. Tools Used for ONDiagnosis

The majority of studies on ON relied on the Bratman’s test (Orthorexia self-test—BOT) [1] and the ORTO-15 questionnaire [39]. Both tools have been translated into several languages and have been used in scientific studies. Recently, alternative methods have been developed such as Eating Habits Questionnaire (EHQ) [40], Düsseldorf Orthorexia Scale (DOS) [12], Teruel Orthorexia Scale (TOS) [41], Barcelona Orthorexia Scale (BOS) [42], and Orthorexia Nervosa Inventory (ONI) [43]. A characteristic of the tools used for ON diagnosis is presented in Table 1.

### 3.2. Orthorexia Self-Test (BOT)

Bratman and Knight [1] developed a 10-item test where the patients are evaluated on the basis of a yes/no scale. The answers were attributed with 1 or 0 points (maximum score = 10). The score of more than four points can point towards the symptoms of ON. The test is a self-evaluation test. Although BOT has not been validated and is not psychometrically valid [1,44], it is used as a diagnostic tool. It was used in the original version by Bundros et al. [17], translated and used in the German [34,45,46], Swedish [47], Polish [48,49,50], and Greek [51,52] studies.

### 3.3. ORTO-15

In 2005,Donini et al. [39] designed a diagnostic tool for ON, which was based on Bratman’s test and Scale 7 of the Minnesota Multiphasic Personality Inventory, MMPI-2 (ORTO-15 is made up of 15 items, which are addressed with Likert 4-dimension scale (never–sometimes–often–always). Each answer was attributed with 1–4 points. The answers pointing towards ON were attributed with 1 point, and those pointing towards healthy eating habits were attributed with 4 points. The final score is the sum of points from 15 items. The lower the scores, the higher the intensity of orthorexicbehaviour [39]. The ORTO-15 scale offers an evaluation of behaviour related to the choice, purchase, preparation, and eating of healthy foods. It distinguishes between three factors relating to eating behaviour: cognitive (items: 1, 5, 6, 11, 12 and 14), clinical (items 3, 7, 8, 9, 15), and emotional (items: 2, 4, 10 and 13). The test items regarding ON symptoms were based on the Bratman’s test (BOT items: 1, 3, 7, 8, 9 and 10), yet some of its verbal aspects have been modified. The ORTO-15 questionnaire has been subject to validation procedures, i.e., the evaluation of diagnostic value of the test (its sensitivity, specificity, and predicative positive and negative value). The study analysed three values of the cut-off point (<35, <40, and <45). ORTO-15 reached satisfactory values for the cut-off points of 40 points (sensitivity = 100%, specificity = 73.6%, positive predicative value = 17.6%, and negative predicative value = 100%) [39]. The quality of the ORTO-15 questionnaire, i.e., its validity and reliability, has not been evaluated. 

It is worth noting that in the validation of ORTO-15 performed by Donini et al. [39], the “wrong group” had the most ON-indicative score. The combination of “healthy” eating behavior and pathological MMPI was supposed to indicate ON in that study, but it was not the group with those features who scored lowest (most ON-like) on the ORTO-15, but rather those with “healthy” eating behavior and normal MMPI (39.4 ± 4 vs. 39.3 ± 4). This result is however not noted or discussed by authors. 

Arusoğlu et al. [32] translated ORTO-15 into Turkish and checked psychometric features of the tool. After a factoranalysis of 15 items of the ORTO-15 questionnaire, the authors chose the items of factor weight ≥ 0.5 for the short test version (ORTO-11) and determined the reliability of ORTO-15 at the Cronbach’s alpha level of 0.44 and 0.62 for ORTO-11. In the following years, other authors adapted ORTO-15 to the country of their study, which led to many versions of the test, e.g., ORTO-12 [5], the Polish version of ORTHO-15 [53], ORTO-11-Hu [54], ORTO-9-GE [55], the English version of ORTO-15 [23,56], ORTO-11-ES [57], ORTO-12-FR [58], and ORTO-6 [59]. These versions differ in terms of the number of items, factors, maximum number of points, cut-off points, and psychometric features. Table 1 shows that the integrity of the tool spans from the unacceptably low value of Cronbach’s alpha of 0.14 [60] to the acceptable value of 0.86 [61]. In order to increase the integrity of ORTO-15, many authors of studies removed its selected items, which changed the tool’s structure. Items such as 1, 2, 8 and 15 were deleted in many studies, which seems to undermine their reliability. According to Dunn et al. [62] the frequency of ON as measured by ORTO-15 is too high. The cut-off point of 40 does not reflect the real prevalence of ON [55]. Therefore, in some studies the cut-off point was lowered to 35 points [63,64], which resulted in a fewer number of cases being diagnosed (Table 1).

Many authors [3,13,15,54,65] question the validity of ORTO-15 due to its limitations, i.e., no clear validation of the tool, no information on the creation of items, no standardisation methods, and an excessive percentage of ON diagnosis in the studied groups.

In some studies, ORTO-15 was translated from English into other languages, e.g., Turkish [20,66,67,68,69,70], Portuguese [71,72,73], Polish [74,75,76,77], Spanish [78,79], Swedish [80], and Arabic [81,82], without any modifications of the tool and with no validation of its quality.

### 3.4. Eating Habits Questionnaire (EHQ)

The EHQ questionnaire was developed in 2013 by D.H. Gleaves, E.C. Graham, and S. Ambwani. It consists of 21 items used to measure knowledge, behaviour, and emotions dealing with an excessive concentration on healthy eating. This tool was developed independently of ORTO-15. The authors developed a three-factor structure of the tool with subscales such as knowledge of healthy eating (5 items), problems with healthy eating (12 items), and positive attitudes towards healthy eating (4 items). EHQ features high integrity (EHQ Knowledge, Cronbach’s alpha = 0.90; EHQ Problems, Cronbach’s alpha = 0.82; EHQ Emotions, Cronbach’s alpha = 0.86). The studied group replies to each item using a 4-point Likert scale (1 = false, not al all true; 2 = slightly true, 3 = mainly true, 4 = very true). The higher the result is, the more likely the diagnosis of ON is [40].

The studies relying on EHQ were performed in the US [83,84,85,86,87,88,89] where the questionnaire was developed and normalised. It should be mentioned that in the English version of the questionnaire, there are two slightly different factor structures for the EHQ questionnaire. Each of two models has three factors; however, in the original model, the first factor is “EHQ Knowledge”, while in the model proposed by Oberle et al. [87], it is “EHQ Behaviour”. What is more, Oberle et al. [87] attributed three items, “I follow a diet with many rules”, “I eat only what my diet allows”, and “I follow a health-food diet rigidly”, to “EHQ Behaviour”, while in the original model, they were attributed to “EHQ Problems” [40]. Such a factor structure was used in later studies [86,88]. In 2018, Brytek-Metera et al. adapted the EHQ questionnaire to the Polish conditions and used it in a study [90]. In 2020, Mohamed Halim et al. [91] developed a 4-factor model of EHQ with new subscales such as EHQ—healthy eating, EHQ—diet restrictions, EHQ—supreme dieting, and EHQ—social impairment. The items attributed to these factors do not meet the content proposed by other authors [40,87]. 

All the authors, despite certain differences obtained in the studies, inform about the high integrity of the tool (Cronbach’s alpha = 0.89–0.9, for particular subscales = 0.7–0.9). According to researchers, EHQ offers a promising psychometric quality [91,92] and can be used to diagnose ON.

### 3.5. Düsseldorf Orthorexia Scale (DOS)

The DOS questionnaire authored by F. Barthels, F. Meyer and R. Pietrowsky, was developed in 2015. There are two versions available: 21-item and 10-item. The longer version of DOS is made up of three subscales: “orthorexic eating behavioir” (10 items), “avoidance of additives” (6 items), and “supply of minerals” (5 items). The shorter version offers only one subscale. The participants use a 4-point Likert scale, fromwith “this does not apply to me” (1 point) to “this applies to me” (4 points). The higher the result, the higher chance of orthorexic behaviour. The cut-off point for the 10-item version is ≥30 points. Both versions demonstrate high integrity (21-item DOS, Cronbach’s alpha = 0.91; 10-item DOS, Cronbach’s alpha = 0.84) [12,24].

The DOS questionnaire was designed for the German-speaking countries. Chard et al. [93] translated the tool into English, which allowed theevaluation of the risk of ON in the English-speaking population and led to the Chinese version of the questionnaire (C-DOS) [94]. 

### 3.6. Teruel Orthorexia Scale (TOS)

The scale was developed in 2018 by J.R. Barrad and M. Roncero. It was designed as a self-evaluation scale, with 4-point Likert scale fromwith “I definitely disagree” (0 points) to “I definitely agree” (3 points). The performed analyses led to the creation of a 17-item tool of a twofold structure. The first factor, a non-pathological interest in healthy eating, known as Healthy Orthorexia (HeOr), is made up of 9 items. The second factor, a pathological dimension of orthorexia (Orthorexia Nervosa—OrNe), is made up of 8 items. Both factors show high reliability. The value of Cronbach’s alpha for HeOr is 0.85, and for OrNe is 0.81. The TOS questionnaire is available in two language versions, Spanish and English [41]. The tool was developed in accordance with the ON concept proposed by Bratman [11]. 

### 3.7. Barcelona Orthorexia Scale (BOS)

The BOS scale was created in 2019 in Spain by S. M. Bauer, A. Fusté, A. Andrés, and C. Saldańa [42]. The tool was developed on the basis of the latest diagnostic criteria by Dunn and Bratman [15] and the available literature on ON. The authors used the Delphi method, which relies on an indirect form of expressing opinions by experts. The participants who formed the panel of experts were researchers and clinicians dealing with eating disorders. Some of them had specialist knowledge on ON, the rest generally specialised in eating disorders. The final BOS version consists of 64 items, in 6 dimensions: cognitive, emotional, behavioral, negative health consequences, negative consequences for social or academic functioning, and differential diagnosis. The basic psychometric quality of the tool was never tested. BOS is also available in Spanish and English [42]. According to our knowledge, there are no studies available that rely on BOS to evaluate ON.

### 3.8. Orthorexia Nervosa Inventory (ONI)

ONI was created by C.D. Oberle, A.S. De Nadai, and A.L. Madrid in 2020 [43]. It consists of 24 items, which need to be addressed on a 4-point Likert scale, beginning from 1 (definitely not true) to 4 (definitely true). ONI is based on the previously designed tools for ON diagnosis, i.e., EHQ and DOS. Some items have been improved in order to effectively differentiate between healthy eating and pathological behaviour. The authors obtained a threefold structure of the tool, with its subscales such as physical and social impairment (ONI impairments—10 items), behaviour and absorption (ONI behaviour—9 items), and emotional stress (ONI emotions—5 items). ONI is the first tool for ON diagnosis whose items evaluate physical impairment. According to scientists and clinicians, it is the key element of the disorder [43]. ONI shows high integrity, with Cronbach’s alpha = 0.94 for the whole tool, and spanning from 0.88 to 0.90 for different scales. It is available in the English language.

**Table 1:** Characteristics of the tools used for ON diagnosis.

Tool	Authors	Year	Country	Number of Items	Structure	Reliability	Responses	Score
**BOT** ***Orthorexia self-test***	Bratman, Knight [1]	2000	USA	10	-	- Psychometric quality (i.e., reliability and validity of the test) has not been established.	A dichotomic format of the responses (yes—1 pts/ no—0 pts)	range: 0–10 ≥4—ON 2–3 pts—tendency for ON
ORTO-15	Donini et al. [8,39]	2004, 2005	Italy	15	Three factors related to eating habits are: − rational—items 1, 5, 6, 11, 12, 14 − clinical—items 3, 7, 8, 9, 15 − emotional—items 2, 4, 10, 13	- Psychometric quality (i.e., reliability and validity of the test) has not been established.	4-point Likert scale (neversometimes–often–always) Reponses pointing towards ON = 1 pts; Responses pointing towards healthy eating habits = 4 pts.	range: 15–60 pts ≤40—ON
ORTO-11	Arusoğlu et al. [32]	2008	Turkey	11; items deleted: 1, 2, 9, 15	One-factor structure of the tool	0.62		range: 0–44 pts
	Fidan et al. [18]	2010			-	-		the cut-off point for ORTO-11 ≤27 pts—ON
ORTO-12	Alvarenga et al. [5]	2012	Brazil	12; items deleted: 1, 2, 15	Threefold structure of the tool: − factor 1—items 3, 7, 11, 13 − factor 2—items 4, 6, 10, 12, 14 − factor 3—items 5, 8, 9	0.39 0.51 0.63 0.47		range 12–48 pts
Polish version of ORTHO-15	Brytek-Matera et al. [53]	2014	Poland	9; items deleted: 1, 2, 8, 9, 13, 15	Twofold structure of the tool: − factor 1—items 4, 5, 6, 10, 11, 12 − factor 2—items 3, 7, 14 Index of two-factor model adjustment: χ^2^ = 35,697 (df = 23, *p* < 0.044); CFI = 0.953; RMSEA = 0.053; PCLOSE = 0.412; AGFI = 0.927	0.644 0.671 0.599		range: 9–36 pts ≤24—ON
	Stochel et al. [95]	2015	Poland	15	-	0.77		range: 15–60 pts ≤ 40—ON ≤35—ON
ORTO-11-Hu	Varga et al. [54]	2014	Hungary	11; items deleted: 5, 6, 8, 14	One-factor structure of the tool; index of one-factor model adjustment: χ^2^ = 230.8; *p* < 0.001; CMIN/DF = 5.63; CFI = 0.92; TLI = 0.90; RMSEA = 0.076; PCLOSE < 0.001.	0.82		range: 11–44 ≤40—ON
ORTO-9-GE	Missbach et al. [55]	2015	Germany	9; items deleted: 1, 2, 8, 9, 13, 14	One-factor structure of the tool; Index of one-factor model adjustment: χ^2^ = 83.865; *p* < 0.001; CMIN/DF = 3.355; CFI = 0.947; TLI = 0.92; RMSEA = 0.048; PCLOSE = 0.602.	0.67		range: 9–36 pts ≤26.7—ON
ORTO-15	Barnes, Caltabiano [23]	2017	Australia	9; items deleted: 1, 2, 8, 9, 13, 15	-	0.73		range 9–36 pts
	Moller et al. [56]	2018	Australia	7; items deleted: 2, 5, 6, 8, 10, 12, 14, 15	One-factor structure of the tool; Index of one-factor model adjustment: χ^2^ =4.9; GFI = 0.97; TLI = 0.94; CFI = 0.96; RMSEA = 0.06;	0.83		range 7–28 pts ≤19—ON
ORTO-11-ES	Parra-Fernandez et al. [57,96]	2018 2018a	Spain	11; items deleted: 5, 8, 14, 15	Three-factor structure of the tool: − rational—items 1, 4, 6, 13 − behavioral—items 2, 3, 7 − emotional—items 9, 10, 11, 12	0.8		range 11–44 pts ≤25—ON
ORTO-12-FR	Babeau et al. [58]	2019	France	12; items deleted: 5, 6, 8	Three-factor structure of the tool: − rational—items 1, 11, 12, 14 − behavioral—items3, 7, 9, 15 − emotional—items 2, 4, 10, 13 Index of three-factor model adjustment: χ^2^ = 144.54, df = 47, *p* = 0.000, CFI = 0.93, TLI = 0.90, RMSEA = 0.05, SRMR = 0.04.	0.73		- the cut-off point for ON has not been established
ORTO-6	Kaźmierczak-Wojtaś [59]	2019	Poland	6; items deleted: 1, 2, 3, 5, 7, 8, 9, 13, 15	-	0.696		ON—6–7 pts tendency for ON—8–11 pts healthy eating—12–15 pts no fixation on eating 16–24 pts
ORTO-10	Mohamed Halim et al. [91]	2020	Australia	items deleted: 1, 2, 8, 9, 13	-	0.76		- the cut-off point for ON has not been established
**EHQ** ***Eating Habits Questionnaire***	Gleaves, Graham, Ambwani [40]	2013	USA	21	Three-factor structure of the tool: − healthy eating behaviours − problems associated with healthy eating − feeling positively about healthy eating Index of three-factor model adjustment: GFI = 0.85; TLI = 0.90; CFI = 0.91; RMSEA = 0.07	EHQ knowledge—0.82 EHQ problems—0.90 EHQ emotions—0.86	4-point Likert scale: 1 = false, not at all true, 2 = slightly true, 3 = mainly true, 4 = very true	the higher the result, the bigger probability of ON.
EHQ	Oberle et al. [87]	2017	USA	21	Three-factor structure of the tool: − healthy eating behaviours − problems associated with healthy eating − - feeling positively about healthy eating	0.9 EHQ behaviours—0.87 EHQ problems—0.79 EHQ emotions—0.73		
EHQ	Brytek-Matera et al. [97]	2018	Poland	21	Three-factor structure of the tool: − healthy eating behaviours − problems associated with healthy eating − feeling positively about healthy eating	EHQ knowledge—0.81 EHQ problems—0.82 EHQ emotions—0.70		
EHQ	Mohamed Halim et al. [91]	2020	Australia	21	Four-factor structure of the tool − factor 1—Healthy Eating Cognitions—items 2, 8, 10, 16, 17, 18 − factor 2 —Dietary Restriction—items 11, 12, 15 − factor 3—Diet Superiority items 3, 7, 9, 13, 14, 19, 21 − factor 4—Social impairment items 1, 4, 5, 6, 20	0.89 EHQ Healthy Eating Cognitions—0.77 EHQ Dietary Restriction—0.72 EHQ Diet Superiority—0.80 EHQ Social impairment—0.77		
**DOS** ***Düsseldorf Orthorexia Scale***	Barthels, Meyer, Pietrowsky [12]	2015	Germany	21 10	Longer version—3 subscales: − orthorexic eating behavior, − avoidance of additives, − supply of minerals Shorter version—1 subscale: − orthorexic eating behavior	0.91 0.84	4-point Likert scale: 1—strongly disagree 2—rather disagree 3—rather agree 4—strongly agree	range 21–84 pts range—10–40 pts ≥30 pts—ON 25–29 pts—risk of ON <25—normal eating behaviours
(E)-DOS	Chard et al. [93]	2019	USA	10	One-factor structure of the tool; Index of one-factor model adjustment: χ^2^ (35) = 216.71, *p* < 0.001; RMSEA = 0.116; GFI = 0.863; AGFI = 0.785; CFI = 0.572	0.882	4-point Likert scale: from “this applies to me“ (4 points) to “this does not apply to me” (1 point)	range—10–40 pts ≥30 pts—ON 25–29 pts—risk of ON <25—normal eating behaviours
C-DOS	He et al. [94]	2019	China	10	Three-factor structure was revealed for the C-DOS; − Obsession in healthy food, − Adherence to strict nutrition rules, − Emotional symptoms. Index of three-factor model adjustment: χ^2^ = 105.16 (df = 32, *p* < 0.01), RMSEA = 0.06 (90% CI 0.05–0.08), CFI = 0.93, TLI = 0.89, SRMR = 0.05;	0.84 0.77 0.75 0.71	4-point Likert scale: “definitely does not apply to me“to “definitely applies to me”	range—10–40 pts ≥30 pts—ON 25–29 pts—risk of ON <25—normal eating behaviours
DOS-ES	Parra-Fernández et al. [98]	2019	Spain	10	-	0.841	4-point Likert scale: 1 = never, 2 = rarely, 3 = often, 4 = always.	range—10–40 pts ≥30 pts—ON 25–29 pts—ON risk <25—normal eating behaviours
**BOS** ***Barcelona Orthorexia Scale***	Bauer et al. [42]	2019	Spain	64	6 areas have been distinguished: − rational; − emotional − behavioral; − negative for health; − negative consequences for social or academic functioning; − differential diagnosis.	-	-	-
**TOS** ***Teruel Orthorexia Scale***	Barrada, Roncero [41]	2018	Spain	17	2-factor model: − healthy orthorexia (HeOr)—9 items (items 1, 2, 3, 6, 7, 8, 11, 13, 15) − orthorexia nervosa (OrNe)—8 items (items 4, 5, 9, 10, 12, 14, 16, 17) Index of two-factor model adjustment: ÷2 (103) = 453.9, CFI = 0.965, TLI = 0.954, RMSEA = 0.060.	HeOr—0.85 OrNe—0.81	4-point Likert scale, from 0 = definitely disagree to 3 = definitely agree	range: HeOr—0–27 pts OrNe—0–24 pts
**ONI** ***Orthorexia Nervosa Inventory***	Oberle, De Nadai, Madrid [43]	2020	USA	24	Three-factor structure of the tool: − physical and social impairment—10 items − behaviour and absorption—9 items − emotional stress—5 items Index of three-factor model adjustment: χ^2^ = 1188.33, *p* < 0.001;	0.94 ONI impairments 0.90 ONI behaviours 0.89 ONI emotions 0.88	4-point Likert scale: “not at all true“ (1), “slightly true“ (2), “mainly true“(3), and “very true“ (4).	range—24–96

AGFI—adjusted goodness of fit index;CFI—comparative fit index;CMIN/DF—Chi-square mean/degree of freedom; GFI—goodness-of-fit index;PCLOSE—*p* (probability) of close fit;RMSEA— root mean square error of approximation;SRMR—standardized root mean square residual;TLI—Tucker–Lewis Index.

### 3.9. Prevalence

The majority of studies on ON prevalence rely on ORTO-15 or one of its adaptations. The studies were carried out mostly in Europe (*N* = 47). Relatively few studies are performed in Australia, Latin America, and North America, where ON has been described for the first time. Table 2 offers a review of the studies, providing the year and country of origin, patients’ group, the tool used, and prevalence of ON.

The indexes of ON prevalence differ depending on the study’s country of origin, patients’ group, and the tool used for ON evaluation. Prevalence of orthorexicbehaviour in the general population as measured by ORTO-15 ranges from 6.9% [62] to 75.2% [82]. In certain groups, the prevalence of ON may reach even 90.6% [99]. In the case of BOT, the prevalence of ON ranges from 0.1% in vegetarians and 0.6% in those following traditional diets [49] to 68.2% in Greek students [52]. In studies relying on DOS, the prevalence of ON ranges from 2.5% in German students [100] to 10.5% in Spanish students [101]. In the case of ONI, the prevalence of ON is 4.5% [43]. The studies relying on EHQ used an inconsistent method of results interpretation; therefore, it is impossible to compare them with other studies. In the case of TOS, the prevalence of ON has not been established. BOS has been described; however, it has not been used in studies. 

Apart from the studies on ON prevalence in general population, specific groups showing a tendency for orthorexic eating behaviour because of their profession (e.g., doctors, dieticians, artists, sports people) or eating habits (vegans, vegetarians) have been studied. What is more, the relationship between socio-demographic factors and ON prevalence has been studied. Some authors believe that ON is more prevalent in men than women [8,18,66,79], while others indicate otherwise [22,25,32,47]. The latest studies undermine these results, pointing towards an equal prevalence of ON among men and women [23,62,102,103,104]. Similar inconsistencies in the literature pertain to age, BMI index, and level of education [32,60,63,66,71,100,105].

## 4. Discussion

An interest in the relatively new phenomenon of ON should lead to an attempt to address thequestion of whether ON is a disorder (e.g., an eating disorder or an obsessive—compulsive disorder) or just a symptom of unhealthy eating behaviour. Not only researchers but also medical staff pay closer attention to those with orthorexicbehaviour, even though neither the American Psychiatric Association nor the World Health Organisation officially acknowledge orthorexia nervosa as a mental disorder. Hence, behaviours characteristic of an excessive fixation on healthy eating should be only treated as a potential disorder.

So far, seven tools for ON evaluation have been developed and described. Some of them, e.g., ORTO-15 (together with its adaptations), have been widely used around the world, while others have been used rarely (e.g., EHQ an TOS) or never (BOS) in formal studies. Each tool has its limitations, identifiedby the authors themselves or other researchers. None of the tools has been used as “a gold standard”, i.e., the most suitable tool for ON evaluation, even though some of them are more promising than others. What is more, there are substantial diagnostic differences between the tools, which suggests that a new concept of diagnostic criteria and, consequently, a construal of a new tool, is needed [129]. 

The indexes of ON prevalence as referred to in the literature differ significantly from those typical of eating disorders, i.e., anorexia nervosa and bulimia nervosa, which are rather rare in the general population [130]. The results obtained by the authors are probably overestimated owing to poor psychometric quality of the ORTO-15 questionnaire [62]. Indexes of ON prevalence show a tendency to a great variability [26,62,67,104], which raises questions regarding the importance and reliability of ORTO-15 for ON evaluation. Taking into consideration other tools such as DOS, the index of ON prevalence does not exceed 8% [93], or, in the case of ONI, does not exceed 4.5% [43]. It should also be noted that ORTO-15 has so many limitations that its use is questionable [3,13,15,54,65]. ORTO-15 is ineffective in diagnosing orthorexicbehaviours and attitudes, and high indexes of ON prevalence are the result of overlapping healhy and orhtorexicbehaviours [15]. Therefore, despite its popularity, it should not be used to evaluate ON.

It should also be noted that the data on ON prevalence are shaped by the validity and reliability of the tools used for its evaluation. The fact that still there is no recommended tool for ON evaluation undermines the estimates of ON prevalence. What is more, owing to a variety of tools used, we should treat the results of the studies with caution.

## 5. Conclusions

A complex analysis of the current state of literature on ON points towards methodological limitations of the empirical studies, which makes it difficult, if possible at all, to draw definite conclusions. An appropriate ON evaluation is a challenge for future studies, as many behaviours fit the norm. Another problem may be a distinction only between those with or without ON, with no diagnosis of those with a tendency for ON. Among the criteria used in the previous studies [10,12, 13,14,15] and psychological factors typical of ON [23,95,119,131], there are certain common areas such as (a) excessive interest in foods (quality, ingredients, effect on health); (b) rigorous eating habits (limiting or eliminating unhealthy foods); (c) perfectionism; (d) a need forcontrol; (e) a feeling of not being understood and socially isolated (social/professional/academic impairment); (f) emotional stress (a feeling of guilt/shame/fear/anxiety); and (g) poor physical health (a drop in nutritional value may lead to malnutrition, loss of body mass, and/or other somatic consequences). A distinction of the group with a tendency of ON, i.e., the group of high risk, is particularly important from the point of view of preventive treatment and education, particularly addressed at this group. A quick diagnosis of eating irregularities can foster an appropriate nutritional attitude and consequently limit the prevalence of ON.

This review is only a part of a bigger research on ON and should be treated as a starting point for further studies.

## Figures and Tables

**Table 2:** Studies on ON prevalence.

Study:			Material:			Methods:		Prevalence (%)
Authors	Year (of Publication)	Country	Studied Group	Number of Patients *NN* (%)		Tool	Reliability (Cronbach’s Alpha)	
Donini et al. [8]	2004	Italy	subjects with various different occupational characteristics	404	F = 236 (41.9) M = 168 (58.1)	ORTO-15	no data	Development of a novel tool for ON diagnosis ORTO-15 range 40 pts Total—6.9 F = 3.9 M = 11.3
Kinzl et al. [34]	2006	Germany	female dieticians	283	F = 283	BOT	no data	Orthorexia nervosa—12.8 Orthorexicbehaviour—34.9
BaǧciBosi et al. [20]	2007	Turkey	resident medical doctors of the Faculty of Medicine	318	F = 149 (46.9) M = 169 (53.1)	ORTO-15	no data	ORTO-15 range 40 pts—45.5
Arusoğlu et al. [32]	2008	Turkey	academic and administrative personel from Hacettepe University	944	F = 578 M = 416	ORTO-11; Deleted items: 1, 2, 9, 15	0.62	Tool adaptation
Aksoydan, Camci [66]	2009	Turkey	performance artists, opera singers, ballet dancers, and symphony orchestra musicians	94	F = 55 M = 39	ORTO-15		ORTO-15 range 40 pts Total—56.4 Opera singers—81.8 Ballet dancers—32.1 Musicians of symphonic orchestra—36.4
Fidan et al. [18]	2010	Turkey	Turkish medical students	878	F = 359 (40.9) M = 464 (52.8)	ORTO-11	0.62	Cut-off points for ORTO-11–27 pts —36.9
McInerney-Ernst [60]	2011	USA	undergraduate students at the University of Missouri-Kansas City (UMKC).	163	F = 58.0 M = 42.0	ORTO-15	0.14	ORTO-15 range 40 pts—83.0 range 35 pts—30.0
Ramacciotti et al. [63]	2011	Italy	general population	177	no data	ORTO-15	no data	ORTO-15 range 40 pts—57.6 range 35 pts—11.9
Alvarenga et al. [5]	2012	Brazil	Brazilian dietitians	392	F = 380 93.0 M = 12 3.0	ORTO-12; Deleted items: 1, 2, 15	0.39	ORTO-12 range 40 pts—81.9
Segura-García et al. [64]	2012	Italy	athletes (taekwondo, boxing, judo, body building, volleyball, basketball, soccer, aerobics, and aqua fitness); 217 sedentary matched controls	577 217	F = 189 M = 388 F = 79 M = 138	ORTO-15	0.81	ORTO-15 range 35 pts F = 28, M = 30
Barthels [24]	2014	Germany (online study)	users of social networks, internet fora, emails	1307	F = 904 M = 393	DOS	0.84	Orthorexia nervosa Total—3.13 F—4.1, M—1.6
Bo et al. [106]	2014	Italy	Students of: − Dietetics, − Biology, − Exercise and Sport Sciences at the University of Turin	440 53 200 187	no data	ORTO-15	no data	ORTO-15 range 35 pts Total—25.9 D = 35.9 S = 26.5 B = 22.5
Brytek-Matera et al. [53]	2014	Poland	men and women, age 18–35 − university students, administrative and teaching personel	400	F = 341 M = 59	Polish version of ORTHO-15; Deleted items: 1, 2, 8, 9, 13, 15	0.64	tool adaptation
de Souza, Rodrigues [72]	2014	Brazil	Nutrition students	150	F = 150	ORTO-15	no data	ORTO-15 range 40 pts —88.7
Herranz Valera et al. [78]	2014	Spain (online study)	ashtanga yoga practitioners	136	F = 89 (65.4) M = 47 (34.6)	ORTO-15	no data	ORTO-15 range 40 pts Total—86 F = 85.5M = 87.2 ORTO-15 range 35 pts Total—43.4 F = 44.9M = 40.4
Neyman et al. [107]	2014	USA	students	448	F = 353 M = 95	ORTO-15	no data	ORTO-15 range 40 pts—81
Varga et al. [54]	2014	Hungary (online study)	students: students from Semmelweis University, EötvösLoránd University, the University of Pécs, and the University of Debrecen.	810	F = 724 (89.4) M = 86 (10.6)	OTRO-11-Hu; Deleted items: 5, 6, 8, 14	0.82	tool adaptation ORTO-11-Hu range 40 pts —74.2
Asil, Sürücüoğlu [67]	2015	Turkey	Turkish dieticians	117	F = 101 (86.3) M = 16 (13.7)	ORTO-15	no data	ORTO-15 range 40 pts —41.9
Barthels et al. [12]	2015	Germany (online study)	1340 people	1340		DOS (10 items)	0.84	orthorexia nervosa—3.0
Brytek-Matera et al. [22]	2015	Poland	women diagnosed with EDs − anorexia nervosa − bulimia nervosa	52 12 40	F = 52	Polish version of ORTHO-15	0.74	Polish version of ORTO-15 range 24 pts —82.7
Brytek-Matera et al. [108]	2015a	Poland	University students of Human Sciences (Psychology and Pedagogy) and Nutrition Sciences (Dietetics) from the Silesia, Lower Silesia, Mazovia, and Lublin Provinces in Poland	327	F = 283 (86.5) M = 44 (13.5)	Polish version of ORTHO-15	0.64	Polish version of ORTO-15 range 24 pts Total—65.1 F = 68.6, M = 43.2
Gubiec et al. [75]	2015	Poland	Polish nutrition students	155	F = 140 (90.3) M = 15 (9.7)	ORTO-15	no data	ORTO-15 range 40 pts —59
Jerez et al. [79]	2015	Chile	High school students	205	F = 94 M = 111	ORTO-15	b.d.	ORTO-15 range 40 pts Total - 30.7 F = 25.5, M = 35.1
Missbach et al. [55]	2015	Germany (online study)	Participants were recruited via online advertisement (social media, email distribution lists) and we collected data online	1029	F = 768 (74.6) M = 261 (25.4)	ORTO-9-GE; Deleted items: 1, 2, 8, 9, 13, 14	0.67	tool validation ORTO-9-GE range 26.7 pts —69.1
Özkan et al. [109]	2015	Turkey	Trakya University Medical School undergraduate students	676	F = 420 (62.1) M = 256 (37.9)	ORTO-11	no data.	Group 1—high risk of ON F = 48.2 M = 51.8 Group 2—medium risk of ON F = 64.4 M = 35.6 Group 3—low risk of ON F = 67, M = 33
Segura-García et al. [29]	2015	Italy	patients diagnosed with EDs: − anorexia nervosa (AN) − bulimia nervosa (BN) − control group (healthy participants)	32 18 14 32	F = 64	ORTO-15	0.81	ORTO-15 range 35 pts clinical group AN—28 BN—53 control group—6
Stochel et al. [95]	2015	Polska	Polish high school students	399	F = 253 (63.4) M = 146 (36.6)	ORTO-15	0.77	ORTO-15 range 40 pts study I—53.7 study II—52.6 ORTO-15 range 35 pts —Total 13.7
Bundros et al. [17]	2016	USA (online study)	a convenience sample of California State University students	448	F = 325 (72.5) M = 121 (27.0) Inne = 2 (0.4)	BOT	no data	healthy eating fixation or orthorexia nervosa F—55.7, M—51.3 Healthy eating F—44.3, M—48.7
Dell’Osso et al. [25]	2016	Italy	students and University employees belonging to University of Pisa	2826	F = 1148 (40.6) M = 1678 (59.4)	ORTO-15	no data	ORTO-15 range 35 pts —32.7
Dittfeld et al. [50]	2016	Poland	Students: − dietetic students − physiotherapy students.	430 229 201	F = 393 M = 37	BOT	no data.	healthy eating fixation D —26.6 F—14.9 healthy eating D—73.4 F—85.1
Farooq, Bradbury [110]	2016	Great Britain	University students who either represented their university competitively in sport or participated for leisure purposes	213	K = 84 (39.0) M = 129 (61.0)	ORTO-15	no data	ORTO-15 range 35 pts Total—37 F= 31 M = 41
Hyrnik et al. [76]	2016	Poland	high school students	1899	K = 992 (52.5) M = 907 (47.8)	ORTO-15	no data	ORTO-15 range 40 pts—61.3 range 35 pts—13.7 range 33 pts—4.2
Sanlier et al. [70]	2016	Turkey	physical and mathematical sciences, and health-related professions	900	K = 522 (58.0) M = 378 (42.0)	ORTO-15	0.71	ORTO-15 range 40 pts —59.8
Arslantaş et al. [111]	2017	Turkey	nursing students	181	K = 141 (77.9) M = 40 (22.1)	ORTO-11	0.64	ORTO-11 range 27 pts —45.3
Barnes, Caltabiano [23]	2017	Australia (online study)	Participants aged 17–62; − first and second year psychology students at James Cook University; − respondents were recruited from Facebook	220 180 40	K = 174 M = 46	ORTO-15 (9 items); Deleted items: 1, 2, 8, 9, 13, 15	0.73	a new version of ORTO-15
Bień, Pieczykolan [74]	2017	Poland	women, age 18–35	280	F = 280	ORTO-15	b.d.	ORTO-15 range 40 pts —71.43
Depa et al. [105]	2017	Germany	students from the University of Hohenheim: − students of nutrition science (NS) − economics (ES) students;	456 188 268	F = 318 (70.0) M = 136 (30.0)	DOS (21 items)	0.91	Orthorexia nervosa Total—3.3 F—2.8 M—3.7 NS students—3.4 ES students—2.1 risk of ON Total—9.0 F—10.4 M—5.9 NS students—11.4 ES students—9.2
Dittfeld et al. [49]	2017	Poland	participants, age 11–70 vegetarians (W) non-vegetarians (Nw)	2611 1346 1265		BOT	no data	orthorexia nervosa W—0.1 Nw—0.6 healthy eating fixation W—30.5 N—26.4 healthy eating W69.5 Nw—73
Dunn et al. [62]	2017	USA (online study)	275 US college students	275	F = 188 (68.0) M = 85 (31.0) Other = 2 (<0.1)	ORTO-15	no data	ORTO-15 range 40 pts—71.2 range 35 pts—22.1
Gramaglia et al. [26]	2017	Poland, Italy	female patients with anorexia nervosa (AN) and healthy controls (HC) from Italy and Poland: − those with anorexia nervosa from Poland − control group from Poland − those with anorexia nervosa from Italy − control group from Italy	136 35 39 23 39	F = 136	ORTO-15	no data	ORTO-15 range 40 pts Poland: AN = 85,6 GrK = 82 Italy: AN = 60,9 GrK = 46
Hayles et al. [112]	2017	USA	undergraduate students at a southeastern U.S. 4-year university.	404	F = 334 M = 70	ORTO-15	no data	ORTO-15 range 40 pts —35.4
Kaźmierczak et al. [113]	2017	Polska (online study)	users of internet fora dedicated to health, eating, and foods	155	F = 136 (87.74) M = 18 (12.26)	ORTO-15 (original and Polish versions)	no data	ORTO-15 range 40 pts —85.16 Polish version of ORTO-15 range 24 pts —78.06
Malmborg et al. [80]	2017	Sweden	undergraduate students	207	F = 117 M = 90	ORTO-15	no data	ORTO-15 range 40 pts—76.6 range 35 pts—26.6
Rudolph et al. [100]	2017	Germany	The sample was recruited among university students who were active members of the university fitness center	759	F = 538 (71.0) M = 221 (29.0)	DOS (10 items)	0.84	orthorexia nervosa Total—2.5 F—2.8 M—1.8
Tremelling et al. [114]	2017	USA (online study)	dieticians	636	F = 615 M = 21	ORTO-15	no data	49.5
Turner, Lefevre [99]	2017	online study -participants mostly from USA and Great Britain	Participants were recruited via not-paid-for advertisements on Instagram, Facebook, and Twitter, as well as the blog “Plantbased Pixie”and the “Heath Bloggers Community“ newsletter	680	F = 680	ORTO-15	no data	ORTO-15 range 40 pts—90.6 range 35 pts—49
Almeida, Vieira Borba, Santos [71]	2018	Portugal	members of two gyms in the city of Coimbra (Portugal)	193	F = 113 (58.5) M = 80 (41.5)	ORTO-15	0.7	ORTO-15 range 40 pts —Total—89.1 ORTO-15 range 35 pts —Total—51.8 F = 48.7 M = 56.3
Andreas et al. [45]	2018	Germany	clinic for Psychosomatic Medicine in Bad Bramstedt	1122	F = 788 (70.0) M = 334 (30.0)	Ortho-10	0.79	tool adaptation
Barthels et al. [115]	2018	Germany (online study)	vegetarians and vegans: − vegans, − vegetarians, − rare meat consumption, − frequent meat consumption; − Sample of dieting individuals − “diet with dietary change“, − “diet without dietary change” − “no diet/control group“	351 114 63 83 91 406 104 37 258	F = 221 (63.0) M = 130 (36.0) F = 322 (79.3) M = 84 (20.7) Inne = 0.2	DOS	0.83	orthorexia nervosa − vegans—7.9 − vegetarians—3.8 − those rarely eating meat—3.6 − those often eating meat—0 − those on a diet—6.7 − those on a diet changing their eating habits—2.7 − control group—1.5
Dell’Osso et al. [116]	2018	Italy	students from the University of Pisa, Italy	2130	F = 1274 (58.9) M = 876 (41.1)	ORTO-15	no data	ORTO-15 range 35 pts —34.9
Gkiouras et al. [51]	2018	Greece	female dietetics students from the Department of Nutrition and Dietetics, in Thessaloniki.	120	F = 120	BOT	no data	orthorexia nervosa—62.9
Grammatiko-poulou et al. [52]	2018	Greece	undergraduate students of the Department of Nutrition & Dietetics, in Thessaloniki, Greece,	176	F = 140 M = 36	BOT	no data	orthorexia nervosa—68.2 F—70.0 M—61.1
Karaçıl Ermumcu, Acar Tek [69]	2018	Turkey	women aged between 20–54 years.	132	F = 132	ORTO-15	no data	ORTO-15 range 40 pts —75.8
Moller et al. [56]	2018	Australia (online study)	social media users, students	585	F = 482 (82.4) M = 103 (17.6)	ORTO-7; Deleted items: 2, 5, 6, 8, 10, 12, 14, 15	0.83	a new version of ORTO-15—range 19 pts Total—34.0 F = 38.6 M = 11.2
Parra-Fernandez et al. [57,117]	2018, 2018b	Spain (online study)	the University of Castilla-La Mancha Spanish University students—Nursing, Law, Chemistry, Computer science and Education;	454	F = 295 (64.98) M = 159 (35.02)	ORTO-11-ES; Deleted items: 5, 8, 14, 15	0.8	tool adaptation and validation ORTO-11 range 25 pts Total—17 F—19.3 M—11.9
Reynolds [104]	2018	Australia	staff and students at the University of New South Wales, Sydney	92	F = 67 (73.0) M = 25 (27.0)	ORTO-15	no data	ORTO-15 range 40 pts—66 range 35 pts—21
Rudolph [118]	2018	Germany	active members of three German professional fitness clubs	1008	F = 449 M = 559	DOS (10 items)	0.84	orthorexia nervosa—4.3 risk of ON—8.8
Strahler et al. [103]	2018	Germany (online study)	people aged 18–75	713	F = 569 (79.8) M = 144 (20.2)	DOS (10 items)	0.87	orthorexia nervosa—3.8
Agopyan et al. [119]	2019	Turkey	female students of the Health Sciences Faculty, Department of Nutrition and Dietetics of a private university in Istanbul	136	F = 136	ORTO-11	0.62	ORTO-11 range 27 pts —70.6
Aslan, Aktürk [68]	2019	Turkey	Women; − patients diagnosed with breast cancer − women who hadnot been diagnosed with cancer	402 238 164	K = 402	ORTO-15	0.79	ORTO-15 range 33 pts patients with breast cancer—23.3 control group = 6.7
Babeau et al. [58]	2019	France (online study)	French individuals, the minimum age was 18 years old, and the maximum age was 85 years old.	768	F = 651 (84.77) M = 117 (15.23)	ORTO-12-FR; Deleted items: 5, 6, 8	0.73	tool validation
Barthels et al. [120]	2019	Germany	− Patients and healthy control group − Patients who were diagnosed with somatoform disorders; − The control group consisted of 30 healthy adults matched with regard to gender, age, and educational levels to the patient sample	61 31 30	F = 17 M = 14 F = 17 M = 13	DOS (10 items)	0.86	orthorexia nervosa patients—6.67 control group—0
Bert et al. [121]	2019	Italy	The sample was recruited among participants (athletes and audience) in local sports events, in particular cyclosportive, running, and walking competitions. No sport Sport <150’/week Sport >150’/week	549 182 47 317	F = 139 (25.5) M = 407 (74.5) b.d. = 3	ORTO-15	no data	ORTO-15 range 40 pts no sport—68.75 Sport <150’/week 71.11 Sport >150’/week 72.76 ORTO-15 range 35 pts no sport—19.89 Sport <150’/week 24.44 Sport >150’/week 21.47 ORTO-15 range 30 pts no sport—1.74 Sport <150’/week 4.44 Sport >150’/week 1.65
Chard et al. [93]	2019	USA (online study)	undergraduate students; Colorado State University;	384	F = 267 (69.5) M = 117 (30.5)	(E)-DOS tool adaptation	0.882	orthorexia nervosa—8.0 risk of ON—12.4 − students following a diet (vegetarianism, veganism, gluten-free diet) orthorexia nervosa—19.4 risk of ON—24.2 − students with no diet orthorexia nervosa—6.0 risk of ON—10.1
Clifford, Blyth [122]	2019	Great Britain	Undergraduate and postgraduate students − student athletes − non-athlete controls	215 116 99	F = 141 M = 74	ORTO-15	no data	ORTO-15 range 40 pts Total—76 F = 75 M = 78
Erkin, Göl [61]	2019	Turkey	Yoga practitioners	118	F = 109 (92.4) M = 9 (7.6)	ORTO-11	0.86	ORTO-11 range 27 pts —75.4
Farchakh et al. [81]	2019	Liban	medical students	627	F = 316 (49.6) M = 311 (50.4)	ORTO-15	0.73	ORTO-15 range 40 pts —74.5
Gorrasi et al. [123]	2019	Italy	Students from: − the University of Turin, − the University of Pavia, − the University of Naples	918 409 202 307	F = (54.8) M = (45.2)	ORTO-15	0.79	ORTO-15 range 35 pts —29.0
Gramaglia et al. [124]	2019	Italy, Poland, Spain (online study)	Students from: − Italy, − Poland, − Spain.	664 216 206 242	F = 400 (72.29) M = 183 (27.71) no data—1	ORTO-15, ORTO-15 Polish version	no data	ORTO-15 range 35 pts Total—37.05 Italy—30.09 Spain—18.18 Polish version of ORTO-15 range 24 pts Poland—66.5
Haddad et al. [82]	2019	Liban	806 community dwelling participantsusing a proportionate random sample from all Lebanese gvernorates (Beirut, Mount Lebanon, North, South, and Bekaa).	806	F = 536 (66.5) M = 270 (33.5)	ORTO-15	0.822	ORTO-15 range 40 pts —75.2
He et al. [94]	2019	China	Students from two universities in mainland China	1075	F = 567 (52.7) M = 508 (47.3)	C-DOS (10 items) tool adaptation	0.8	orthorexia nervosa Total—7.8 F—5.3 M-10.6 risk of ON—18.2 F—14.5 M—22.4
Heiss et al. [125]	2019	USA (online study)	participants were recruited via Facebook pages focused on vegetarianism and veganism, and other websites about food, psychology, and psychological research. omnivore meat reducer lacto-ovo-vegetarian vegan	381 106 34 50 191	F = 308 (80.8) M = 73 (19.2)	ORTO-15	0.30–0.42 mixed dieting = 0.30 vegetarians = 0.39 lacto-ovo-vegetarians = 0.42 vegans = 0.37	ORTO-15 range 40 pts 77.7
Kaźmierczak-Wojtaś [59]	2019	Poland	young people aged 16–35	473	F = 331 (70.0) M = 142 (30.0)	ORTO-6	0.696	ON—range 6-7 pts Total– 3.6% F = 4.2 M = 2.1 risk of ON—range 8-11 pts Total—29.2 F = 30.5 M = 26.1
Luck-Sikorski et al. [102]	2019	Gernany (telephone interview)	the German general public	1007	F = 489 (48.6) M = 518 (51.4)	DOS (10 items)	0.80	orthorexia nervosa Total—6.9 F—7.9 M—5.9
Łucka et al. [126,127]	2019, 2019 a	Poland	school-age youth and young adults from Pomeranian and Warmian-Masurian voivodeships.	864	F = 599 M = 265	ORTHO-15	no data	ORTO-15 range 40 pts—76.7 range 35 pts—27.8
Parra-Fernández et al. [101]	2019a	Spain	students from Casilla la Mancha University, Spain	492	F = (56.9) M= (43.1)	ORTO-11-ES DOS-ES	0.84 0.79	ORTO-11-ES range 25 —25.2 DOS-ES—range 30 pts —10.5
Plichta, Jeżewska-Zychowicz [77]	2019	Poland	participants recruited from seven universities in Poland.	1120	F = 789 (70.4) M = 331 (29.6)	ORTHO-15	0.7	ORTHO-15 range 40 pts—75 range 35 pts—28.3
Oberle et al. [43]	2020	USA (online study)	Texas State University students and social media users (Facebook, Instagram)	847	F = 692 (82.0) M = 125 (18.0)	ONI	0.94	ONI—range 72 pts —4.5
Plichta, Jeżewska-Zychowicz [128]	2020	Poland	Polish students	1120	F = 789 (70.4) M = 331 (29.6)	ORTO-15	0.7	ORTO-15 range 35 pts —28.3

F—female; M—male; BOT—Orthorexia self-test; ORTO-15—The ORTO-15 questionnaire; DOS—Düsseldorf Orthorexia Scale; ONI—Orthorexia Nervosa Inventory.

## Data Availability

The data presented in this study are openly available in PubMedCentral (PMC) and Google Scholar.
